# Factors Affecting the Receptiveness of Chinese Internists and Surgeons Toward Artificial Intelligence–Driven Drug Prescription: Protocol for a Systematic Survey Study

**DOI:** 10.2196/76009

**Published:** 2025-08-14

**Authors:** Qiujin Shen, Xiaowen Gong, Wei Zhang, Yu Hu, Yueshen Ma, Ziyi Ren, Sichang Liu, Zhen Song, Robert Peter Gale, Jinyu Wang, Junren Chen

**Affiliations:** 1 State Key Laboratory of Experimental Hematology National Clinical Research Center for Blood Diseases, Haihe Laboratory of Cell Ecosystem, Institute of Hematology & Blood Diseases Hospital Chinese Academy of Medical Sciences & Peking Union Medical College Tianjin China; 2 Tianjin Institutes of Health Science Tianjin China; 3 Centre for Haematology, Department of Immunology and Inflammation Imperial College of Science, Technology and Medicine London United Kingdom

**Keywords:** artificial intelligence, conditional autonomy, prescription, receptiveness, systematic survey

## Abstract

**Background:**

Recently, we developed and tested an autonomous artificial intelligence (AI) agent for prescribing a drug to prevent severe acute graft-versus-host disease in patients receiving human leukocyte antigen haplotype–mismatched hematopoietic cell transplants in a prospective clinical trial. Our experience in this proof-of-concept study suggests that physicians and patients can be receptive to autonomous AI prescription. However, the generalizability of our conclusion requires testing in additional clinical settings. Before broadening the scope of study of AI-driven drug prescriptions, it is important to quantify the factors that influence a physician’s receptiveness to AI prescription.

**Objective:**

We aim to systematically interrogate physicians’ receptiveness to AI prescription in China.

**Methods:**

We have designed a research protocol to survey a diverse range of factors that may affect physicians’ receptiveness to AI prescription systems, including the physicians’ personal attributes and their perceptions of the importance of various technological, institutional, and governmental attributes. The survey will be conducted in 2 phases. In phase 1, the survey will be limited to the Tianjin metropolitan area, enlisting >250 physicians from approximately 2 tier-1, 3 tier-2, and 3 tier-3 hospitals. In phase 2, we will survey metropolitan areas in ≥10 additional province-level administrative divisions, enlisting >1250 additional physicians from >15 tier-1, >15 tier-2, and >15 tier-3 hospitals. We hypothesize that physicians can be broadly classified into distinct psychological profile types, and furthermore, that these types are plausibly mediated by the locales where the physicians are employed and the physicians’ demographics, educational and job experience, clinical subspecialties, and previous knowledge of and experience with AI. Clustering methods, including *t-*distributed stochastic neighbor embedding and hierarchical clustering, will be performed on respondent data to identify the distinct psychological profile types of the physicians. Multiple-variable regression and mediation analyses will be conducted to identify potential underlying mechanisms mediating physicians’ receptiveness to AI prescription.

**Results:**

At the time of submission of the manuscript, no subjects have been recruited. The survey study was approved by the institutional ethics committee and funded in May 2025, and we started recruiting respondents in May 2025. We plan to complete phase 1 by September 30, 2025, and phase 2 by November 30, 2025. Anonymized survey results and their analyses are expected to be published in a peer-reviewed journal in fall 2026.

**Conclusions:**

We anticipate that data and analytical insights generated from this study will assist policy makers and AI researchers in prioritizing a data-informed sequence of developing and promoting AI prescription tools in successive regions, disciplines, and clinical use cases and inform policy makers to match resource allocation with “AI readiness.”

**International Registered Report Identifier (IRRID):**

PRR1-10.2196/76009

## Introduction

### Background

There is growing interest in using autonomous artificial intelligence (AI) in clinical practice [1]. However, most prospective trials of autonomous AI have investigated image-based diagnosis, radiation therapy planning, behavioral nudges, or chatbots, rather than drug prescriptions [2-16]. Previously, AI models for recommending drug prescriptions have been constructed, but they have rarely been used prospectively in clinical settings [17,18]. Recently, we developed and tested an autonomous AI agent for prescribing a drug to prevent severe acute graft-versus-host disease in patients receiving human leukocyte antigen haplotype–mismatched hematopoietic cell transplants, in a prospective clinical trial (ClinicalTrials.gov [NCT05600855]) [19,20]. Our proof-of-concept study suggested that both physicians and patients can be receptive to autonomous AI prescription in the studied setting. However, the generalizability of our conclusion requires testing in additional clinical settings. We submit that, before broadening the scope of studying autonomous AI in drug prescription, it is important to systematically canvass and quantify potential factors that might influence a physician’s receptiveness to using autonomous AI in drug prescription.

### Objectives

We hypothesize that physicians can be broadly classified into distinct psychological profile types and that these psychological profile types are at least partially mediated by the locales where the physicians are employed and the physicians’ demographics, educational and job experience, clinical subspecialty, and previous knowledge of and experience with AI. Here, we report a survey protocol that we have designed to systematically interrogate physicians’ receptiveness to AI prescription in China.

## Methods

### Ethical Considerations

The proposed survey study has been approved by the ethics committee (QTJC2025047-EC-1) at the Institute of Hematology, Chinese Academy of Medical Sciences (IHCAMS) on May 15, 2025. Only respondents who provide informed consent with e-signatures will be granted access to the survey website. All entered data will be stored in password-protected secure servers. All data will be anonymized before analyses, and no write-up of survey results resulting from this study will contain information that can be used to identify any respondent. No incentives—either monetary or nonmonetary—will be offered to participants taking part in the survey.

### Questionnaire Design

Lists of covariates that may affect physicians’ acceptance of medical AI, including physicians’ personal attributes and perceptions of the importance of self-autonomy, multiple technology attributes, institutional attributes, and governmental attributes, have been compiled in the literature [21-24]. Social influence, perceived ease of use, computer anxiety, and other factors may also play important roles [25-27].

Building on the foundation of previous work related to technology acceptance, we have designed a questionnaire (Multimedia Appendix 1 [English translation] and Multimedia Appendix 2 [original Chinese version]) to interrogate physicians’ receptiveness to AI prescription, encompassing 4 dimensions of covariates (Table 1).

**Table 1:** Surveyed covariates^a^.

Dimensions and covariates			Questions
**Personal attributes**			
	Demographics	Q1. Birth year Q2. Sex Q3. Highest educational degree Q4. Clinical practicing experience Q5. Current rank in the professional ladder Q6. Clinical specialty	
	Knowledge of AI^b^	Q7. I am knowledgeable in AI technology in general. Q8. I am knowledgeable in medical AI technology. Q9. I am experienced with using AI in work and daily life. Q10. I am experienced with using medical AI in my clinical practice.	
	Perception of AI	Q11. I believe AI will eventually transform the health care industry. Q12. I believe AI will eventually augment physicians’ capabilities. Q13. I believe AI will eventually improve health care quality. Q14. I believe AI will eventually improve health care equity. Q15. I believe medical AI will eventually facilitate physician training. Q16. Which is more important to me: AI efficacy versus my autonomy?	
	Perception of AI prescription	Q17. I believe AI prescription might be useful in ____ situations.	
**Perceived importance of technological attributes**			
	Vetted efficacy	Q18. I think an AI prescription model’s efficacy needs to be vetted in _____ manners.	
	Expediency	Q19. I think an “expedient” AI prescription model needs to have _____ properties.	
	Transparency	Q20. I think technical details regarding how an AI prescription model was built and validated need to be transparent before I start using the model in my own clinical practice.	
	Explainability	Q21. I think an “explainable” AI prescription model needs to have _____ properties.	
	Governance and stewardship	Q22. I think ____ aspects of governance and stewardship are the most important for successful adoption of an AI prescription model in my own clinical practice.	
	Trade-offs	Q23. Rank the perceived importance of these technological attributes: (1) vetted efficacy, (2) expediency, (3) transparency, (4) explainability, and (5) governance and stewardship.	
**Perceived importance of institutional attributes**			
	Culture	Q24. I think ___ aspects of institutional culture will be the most crucial for successful adoption of AI prescription.	
	Change management	Q25. I think ___ aspects of change management will be the most crucial for successful adoption of AI prescription at my institution.	
	IT and data science	Q26. I think ___ aspects of information technology and data science resource availability and access will be the most crucial for successful adoption of AI prescription in my clinical practice.	
	Differentiation	Q27. I think my institution will need to improve ____ to maintain or further its edge compared to other medical institutions after adoption of AI prescription systems.	
**Perceived importance of governmental attributes**			
	Nation-wide commitment	Q28. I think ____ aspects of governmental policy will be the most crucial for successful adoption of AI prescription in my clinical practice.	
	AI standards	Q29. I think ____ should set the standards for AI prescription systems.	
	Remuneration policy	Q30. I think ___ should be remunerated when an AI model is used to prescribe a drug.	
**To sum up**			
	—^c^	Q31. I anticipate ≥1 physician (possibly, including myself) at my hospital will become ready to use AI to prescribe a drug within _____ years. Q32. I anticipate I myself will become ready to use AI to prescribe a drug within _____ years.	

^a^The abbreviated version of the list of surveyed covariates is displayed here. The full questionnaire that will be used for conducting the survey is provided in Multimedia Appendices 1 and 2.

^b^AI: artificial intelligence.

^c^Not applicable.

The survey questions are structured under each of the 4 dimensions. The first dimension includes the physician’s personal attributes, including demographic information (eg, age, sex, education level, clinical practicing experience, seniority, and clinical subspecialty); self-assessed level of confidence in making informed judgments regarding AI technologies; self-assessed level of experience with using AI in work and daily life; self-assessed probability of the success of using medical AI to induce beneficial transformation of the health care industry, augment physicians’ patient care capability, improve equity in health care, and facilitate physicians’ training; and the physician’s attitude toward the trade-off between AI model efficacy and the sanctity of the physician’s own autonomy.

The second dimension includes the physician’s perceived importance of various aspects or attributes of AI technology, including how an AI prescription model’s clinical efficacy should be vetted and which organizations are endorsing the AI model; how the AI model might impact clinical workflows, workload, throughput, cost-effectiveness, risk of making errors, and physician-patient relationship; whether the AI model’s technical details are transparent and readily accessible to the physician; whether the physician fully understands and agrees with the model’s input variables, input-output relationship (ie, the underlying algorithm), the reasoning or justification behind the model’s drug prescription, and how easy it is for the physician to explain the model’s mechanistic details to their colleagues and patients; and how stewardship of the AI prescription model would be administered after the model’s initial deployment in clinical settings, particularly, regarding model upgrades, safety monitoring, and data privacy and security.

The third dimension includes the physician’s perceived importance of various institutional attributes, including workplace culture (eg, whether the institution encourages introduction of new technology and values innovation, interpersonal relationship, team collaboration, transparent communication, talent development, result orientation, and agility); mechanisms for catalyzing changes in the organization (eg, an enlightened leadership team, a dedicated team of local champions “owning” the implementation of new AI technologies, a collaborative and transparent modus operandi of implementing new technologies within the organization, or an on-boarding plan for educating and training staff members to leverage new technologies); in-house IT expertise; access to third-party IT expertise; and how the institution should or could differentiate itself from other medical institutions once new AI technologies are introduced.

The fourth dimension includes the physician’s perceived importance of various governmental attributes, such as governmental policies to encourage exploration of new use cases for AI in clinical medicine, governmental efforts to improve technological infrastructure for medical AI, governmental commitment to investment in educational programs cultivating new talents specializing in medical AI, publicization and institution of governmental standards for use of AI for drug prescription, and reimbursement policies for using an AI prescription model.

At the end of the survey, we will ask the respondents to answer 2 forced-choice questions (Q31 and Q32), as presented in Textbox 1.

The questionnaire has been tested internally on 20 physicians at the IHCAMS and modified and improved based on their feedback.

**Forced-choice questions in the survey questionnaire.**

**I anticipate ≥1 physician (possibly, including myself) at my hospital will become ready to use AI to prescribe a drug within _____ years.**

- ≤1
- 1.1 to 3
- 3.1 to 5
- 5.1 to 10
- >10
- Never

**I anticipate I myself will become ready to use AI to prescribe a drug within _____ years.**

- ≤1
- 1.1 to 3
- 3.1 to 5
- 5.1 to 10
- >10
- Never

### Stratified Sampling of Metropolitan Areas, Hospitals, and Physicians

To ensure that survey takers will be a balanced representation of the physician population in China, the survey will not be open or based on a convenience sample. Rather, we will conduct a stratified sampling of Chinese physicians.

Setting type-I error α=.025 and type-II error β=.1 (without correction for multiple-hypotheses testing), to achieve 90% power to observe the squared correlation coefficient ρ^2^>0.1 between independent covariates (with up to 120 df) and the 2 dependent covariates (Q31 or Q32) while the true value of ρ^2^ is 0.16, we estimated that ≥1515 physicians will need to participate in the survey.

**Figure 1:**
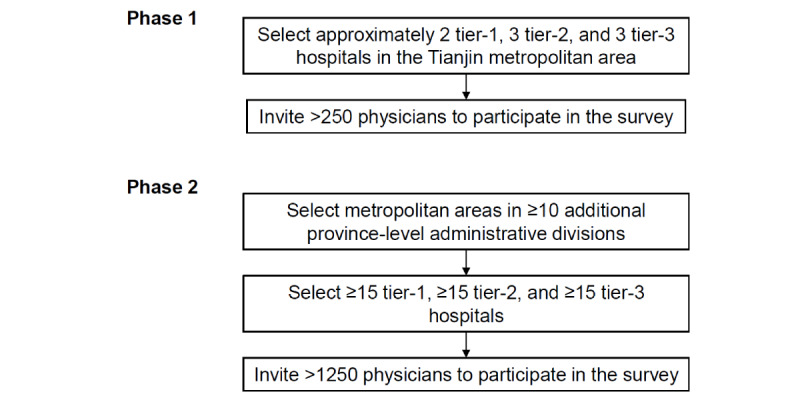
Stratified sampling of metropolitan areas, hospitals, and physicians.

We plan to conduct the survey in a diverse set of metropolitan areas in >10 of the 31 province-level administrative divisions of the Chinese mainland, with diversity defined in terms of per capita gross domestic product and geography (Figure 1). For each sampled metropolitan area, we will conduct the survey in representative tier-1 (*yiji*; lowest level), tier-2 (*erji*), and tier-3 (*sanji*; highest level) hospitals (a tier-1 hospital in the Chinese mainland is equivalent to a community clinical service center in the United States, while a tier-2 hospital is a midsized hospital and a tier-3 hospital is a larger-sized hospital or an academic medical center). Hospitals with higher patient volumes will be preferentially selected.

For each selected hospital, we will sample a diverse subset of internists and surgeons. Physicians invited to participate in the survey will represent the full range of clinical subspecialties within internal medicine or surgery and the 3 successive seniority ranks, including “chief physician” (*zhuren yishi*), “associate chief physician” (*fuzhuren yishi*), and “junior attending physician” (*zhuzhi yishi*).

The survey will be administered in 2 phases.

In phase 1, the survey will be limited to Tianjin, a municipal administrative area adjacent to Beijing with 13.6 million residents [28] and under direct administration of the central government of the People’s Republic of China. We anticipate surveying approximately 2 tier-1, 3 tier-2, and 3 tier-3 hospitals, enlisting >250 physicians. This pilot study will allow us to fine-tune the logistics and survey administration before rolling out the survey to additional metropolitan areas. We do not anticipate modifying the questionnaire based on experience in phase 1.

In phase 2 of the study, we will survey metropolitan areas in ≥10 additional province-level administrative divisions, with a balanced representation of the Pacific coast (which is economically better developed) and less developed regions, and collect returned questionnaires from >1250 physicians from ≥15 tier-1, ≥15 tier-2, and ≥15 tier-3 hospitals.

### Contact Mode and Privacy Protection

For each sampled hospital, the research team will request the hospital administration to assist in the sampling of their internists and surgeons and to make initial contact with the potential participants on behalf of the research team. The hospital administration will be instructed to ensure that the physicians invited to participate in the survey are a balanced representation of the range of clinical subspecialties and the 3 successive seniority ranks. Participation will be entirely voluntary (the Chinese Academy of Medical Sciences will have no authority over promotion, bonus decisions, or any other aspect of personnel evaluation at any of the sampled hospitals). No incentives—either monetary or nonmonetary—will be offered to the physicians agreeing to participate in the survey.

Once the administration of the sampled hospital identifies a date or dates for survey taking, the research team will dispatch representatives to be physically present at survey-taking sessions to serve as moderators, whose primary goal will be to ensure that there will be no technical errors or misunderstanding of the survey questions. Therefore, the respondents will not be anonymous, in the sense that the hospital administration will know who will attend the survey-taking sessions and that the moderators will see the respondents in person, unlike in most e-surveys wherein the identity of respondents is unknown to the researchers. Nevertheless, the identity of the respondents will be strictly protected in this study. Only anonymized data that cannot be used to identify the respondents will be used for data analyses. Furthermore, when the survey results are disseminated, the respondents will remain anonymous in accordance with the directive of the IHCAMS Ethics Committee.

### Informed Consent and Survey Administration

Each separate survey-taking session will have a separate survey website hosted on Alibaba Cloud secure servers. Each separate survey website will have its own preset live period and expiration time. All survey-taking sessions will use an identical user interface, which was developed by the authors.

**Figure 2:**
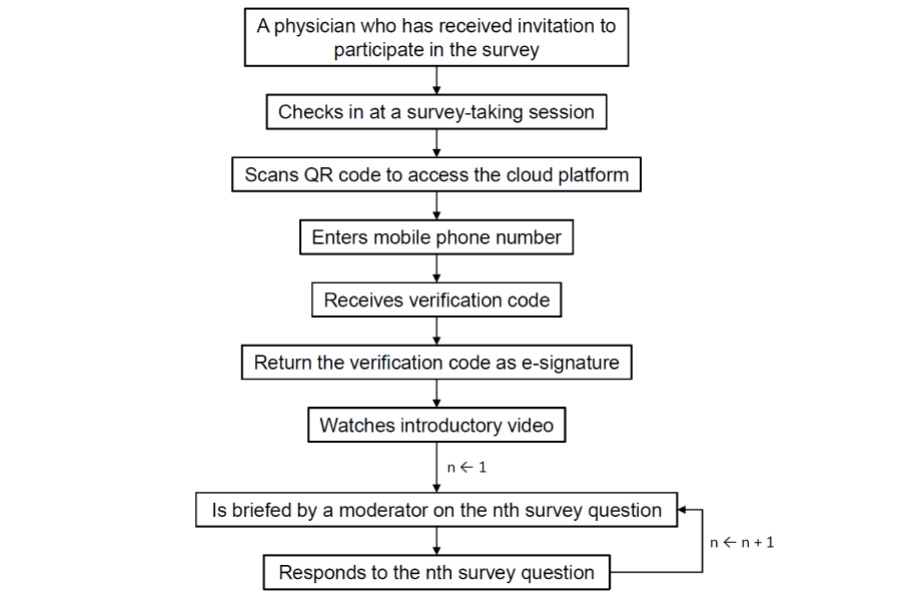
Procedure for participating in the survey.

Respondents in a survey-taking session will first scan a QR code with their personal smartphones to access the survey website. Once connected, the respondents will be prompted to enter their mobile phone number. The message “I understand the purpose of this survey and agree to participate” will be displayed on the phone screen. Concurrently, individualized verification codes will be sent to the respondents via SMS text messages, and to confirm their consent to participate in the survey, respondents will reply with the code (Figure 2). Each phone number can be used only once for participating in the survey. Because “burner” devices are uncommon in the Chinese mainland, the verified phone number will serve as the e-signature for each respondent. The verified phone numbers are protected and will not be used for future communications with the respondents. In other words, the researchers will not call any of the respondents during the follow-up analyses. All entered data, including phone numbers and answers to survey questions, will be stored in password-protected secure Alibaba Cloud servers, and data access will be limited to 4 of the coauthors.

**Figure 3:**
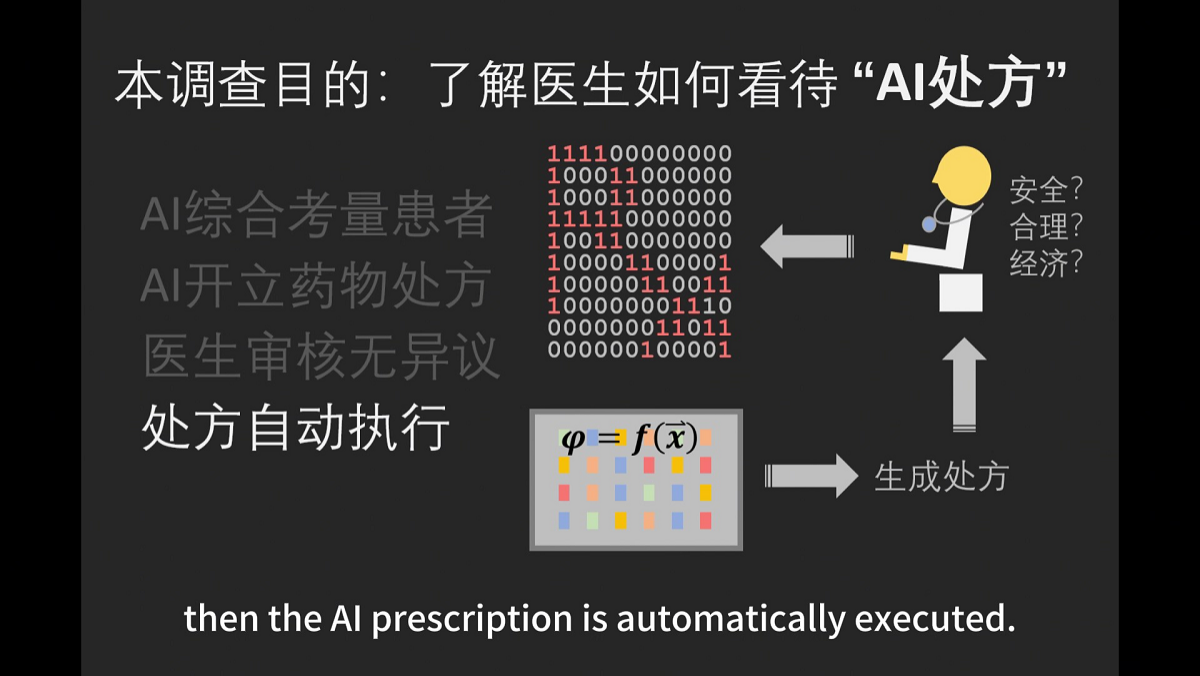
Screenshot of the introductory video. The title is “Purpose of this study: to understand physicians’ attitudes toward AI prescription”. The text on the left translates (from top to bottom) to “AI evaluates a patient based on multimodal data”; “AI prescribes a drug or drugs”; “The physician does not veto after reviewing”; and “The prescription is executed by default”. The text on the right translates (from bottom to top) to “Generating a prescription”; “Safe? Reasonable? Economical?” AI: artificial intelligence.

After providing their informed consent, respondents first watch a 50-second-long video (Figure 3 [screenshot] and Multimedia Appendix 3 [original video with English captions]) to be reminded of the purpose of the survey, with the following script:

As artificial intelligence (AI) technology progresses, more tasks are executed or assisted by AI. For example, drafting of a press release, automated parking, image-based diagnosis, and surgical robots. According to the 15th Five-Year Plan, China will focus on AI+ for national development, in particular, exploring new use cases for AI. This survey aims to understand physicians’ attitudes towards AI prescription. “AI prescription” entails the following steps: First, AI considers multiple factors of a patient; then, AI prescribes medication based on a pretrained model. If the physician thinks the AI prescription is safe, reasonable, and economical, then the AI prescription is automatically executed. AI prescription might not be without risk, but it might also unleash new productivity gains. Your inputs are highly valued.

The 15th Five-Year Plan is a national economic development plan of the People’s Republic of China for 2026-2030 that will emphasize AI and is known to most Chinese residents, including physicians. As of the time of writing this paper, the 15th Five-Year Plan has not spelled out a detailed road map for using AI in clinical practice, and the phrase “AI prescription” is not mentioned in official statements.

Afterward, respondents will proceed to take the survey (Figure 1). Each page will display only one question. Moderators will be present to explain the intent of a survey question if the respondents request clarification. Items in multiple-choice questions will not be randomized, and we will not scramble the order of the questions for different respondents because many questions are Likert-scale questions, and there is a natural progression of the themes asked by the questions. Survey questions will not be “adaptive,” and each respondent will be expected to answer all the survey questions. Proper answering of forced-choice rank-order questions will be enforced by the survey website (eg, if a respondent chooses more than 3 items when they cannot select more than 3, they will not be able to submit their answer). The respondent’s answers to survey questions are stored sequentially and cumulatively, and if they accidentally exit the survey before completing it, they can reenter the survey using their mobile phone number (with SMS code verification), and their previous data entries will be automatically restored. The respondent cannot click “Final submission” unless they answer all the survey questions. The respondent will be able to switch between questions and modify their answers as long as they have not clicked “Final submission.” However, once “Final submission” has been clicked, the respondent cannot reenter the survey to modify their answers.

### Analyses

Each unique respondent will be distinguishable by their unique mobile phone number. The completion rate will be quantified by the number of people who click “Final submission,” divided by the number of people who provide informed consent. Questionnaires filled out in too short a time will be treated as bad responses and excluded from further analyses. Bad response rates will be reported. A balanced representation of hospitals in different tiers and physicians with different specialties and levels of experience will be confirmed by inspection of respondent statistics.

Answers to all survey questions will be converted to numerical values before analyses. Responses to Likert-scale questions will be converted from 5 (highly agree) to 1 (highly disagree). Responses to forced-choice rank-order questions will be coded in two ways: (1) a respondent’s first-, second-, and third-choice items will be coded 3, 2, and 1, respectively (if the question asks the respondent to select their ≤3 top choices), and unselected items will be coded 0; (2) alternatively, all the selected items of a respondent will be coded 1 regardless of their rank orders, while all the unselected items will be coded 0.

One of our main hypotheses is that physicians can be broadly classified into distinct psychological profile types characterized by distinct patterns of their answers to Q11 to Q32, and clustering methods, including *t*-distributed stochastic neighbor embedding and hierarchical clustering, will be used to uncover the psychological profile types. We anticipate that distinct types of physicians will have different criteria for evaluating an AI model (eg, one type of physicians may think they need to be able to explain to their patients the inner workings of an AI prescription model before they are willing to adopt it in their clinical practice) and different clinical needs (eg, one type of physicians might particularly welcome the use of AI prescription to expedite outpatient care of persons with chronic diseases who require periodic prescription refills, while another type might think that AI prescription models are the most useful for early intervention of adverse events in complex situations, such as hematopoietic cell transplantation), and—most importantly—different psychological profile types of physicians may have different levels of receptiveness to AI prescription. Geospatial analysis will be performed to map the composition and variance of psychological profile types across the metropolitan areas and provinces.

Our second main hypothesis is that physicians’ psychological profile types are at least partially mediated by the locales where they are employed and their demographics, educational and job experience, clinical subspecialty, and previous knowledge of and experience with AI, characterized by their answers to Q1 to Q10. Multiple-variable regression and mediation analyses will be conducted to infer the potential mechanisms underlying the formation of distinct psychological profile types. For example, suppose we learn that clinical subspecialty X and geographic region Y are strong mediators of high receptiveness to AI prescription; then, we could be confident in prioritizing the development of AI prescription tools serving subspecialty X in geographic region Y.

Robustness of analytical results will be assessed via bootstrapping (ie, sampling with replacement).

### Dissemination Plan

Anonymized survey results and their analyses will be published in a peer-reviewed journal. Separate analytical reports that continue to uphold respondent privacy but will focus on subsets of hospitals in subsets of regions may also be produced and disseminated to select hospital administrations for more targeted, locally focused considerations. Respondent identity will not be disclosed in any—published or unpublished—report.

## Results

We plan to complete phase 1 of the survey by September 30, 2025, and phase 2 by November 30, 2025. Analyses of the survey results will be completed by January 31, 2026.

## Discussion

### Anticipated Findings

We anticipate that our survey will reveal that a sizable portion of physicians in China are receptive to AI prescription and, furthermore, that in-depth analyses will shed light on potential mechanisms underlying their AI optimism. This knowledge, in turn, can inform us how to “nudge” physicians that are less receptive to AI prescription to become more receptive. Lessons learned from the resultant “map” of Chinese physicians’ minds or psychological profile types will be a useful addition to the medical AI literature.

However, we caution that the lessons learned from our China-focused study might not extrapolate to physicians in other countries. Many people in BRICS (Brazil, Russia, India, China, and South Africa) countries (representing approximately a quarter of the world economy) are considerably more receptive to medical AI and more willing to trust in medical AI systems compared with people in other countries, such as France, Germany, Japan, the United Kingdom, and the United States [29]. Because there is wide variance in receptiveness to medical AI across different countries, it will be imperative to interpret the results of our China-focused survey from a global perspective.

The data collected in the proposed survey study will allow us to construct high-granularity profiles of respondents’ psychology with respect to receptiveness to AI prescription. For example, one plausible outcome might be that physicians are broadly classified into 2 psychological profile types, optimists and pessimists, with the former more receptive to the use of AI prescription in their clinical practice while the latter more resistant to the adoption of AI prescription. Physicians with different profile types might have different criteria for evaluating the acceptability of an AI agent. For example, one profile type might value clinical efficacy, while the other profile type might emphasize the sanctity of their own autonomy or job security. Different profile types might also have different expectations for their employers’ role in promoting AI prescription. For example, one profile type might think it is of utmost importance that the hospital invests heavily in AI infrastructure while the other profile type might believe it is more important that the hospital administers a staff training program for AI use.

We also anticipate using the survey data to identify the key mediators that underlie the variance in physicians’ receptiveness to AI prescription. For example, survey data may reveal that physicians who already have on-the-job experience with medical AI have less AI anxiety and higher receptiveness to AI prescription. Alternatively, it is also plausible that it is the locale where the physician is employed (eg, a tier-3 hospital in an economically developed urban area vs a tier-1 hospital in a less developed region) that predominantly determines their receptiveness. We also anticipate learning that some mediators of receptiveness to AI prescription are more modifiable than others. For example, suppose survey data indicate that previous knowledge of AI is a very strong mediator of receptiveness; then, we can potentially shift the distribution of psychological profile types of physicians toward higher receptiveness by investing in graduate-level and postgraduate educational and training programs that will familiarize more medical students and physicians with AI and the science behind it. Identification of these enablers and blockers for technology adoption will be instructive for prioritizing a data-informed sequence of developing and promoting AI prescription tools in successive regions, disciplines, and clinical use cases, and inform policy makers to match resource allocation with AI readiness.

### Future Directions

We anticipate that future directions after the conclusion of the survey study will encompass 2 important parallel paths. First, it will be imperative to incorporate AI and additional novel informatics tools in clinical practice to further reduce human errors and improve patient care whenever and wherever there is AI readiness [30,31]. Second, we will champion a research program focusing on when it is appropriate and beneficial to “nudge” physicians toward higher AI readiness in treatment decision-making [32,33].

## Appendix

Multimedia Appendix 1 Questionnaire (English translation).

Multimedia Appendix 2 Questionnaire (original Chinese version).

Multimedia Appendix 3 Introductory video.

## Abbreviations

AI: artificial intelligence
BRICS: Brazil, Russia, India, China, and South Africa
IHCAMS: Institute of Hematology, Chinese Academy of Medical Sciences

## Data Availability

Anonymized survey data will be made available to other researchers upon request in accordance with the People’s Republic of China regulations on data privacy and security.
