# Correction: Factors Affecting the Receptiveness of Chinese Internists and Surgeons Toward Artificial Intelligence–Driven Drug Prescription: Protocol for a Systematic Survey Study

**DOI:** 10.2196/83537

**Published:** 2025-09-10

**Authors:** Qiujin Shen, Xiaowen Gong, Wei Zhang, Yu Hu, Yueshen Ma, Ziyi Ren, Sichang Liu, Zhen Song, Robert Peter Gale, Jinyu Wang, Junren Chen

**Affiliations:** 1 State Key Laboratory of Experimental Hematology National Clinical Research Center for Blood Diseases, Haihe Laboratory of Cell Ecosystem, Institute of Hematology & Blood Diseases Hospital Chinese Academy of Medical Sciences & Peking Union Medical College Tianjin China; 2 Tianjin Institutes of Health Science Tianjin China; 3 Centre for Haematology, Department of Immunology and Inflammation Imperial College of Science, Technology and Medicine London United Kingdom

In “Factors Affecting the Receptiveness of Chinese Internists and Surgeons Toward Artificial Intelligence–Driven Drug Prescription: Protocol for a Systematic Survey Study” (JMIR Res Protoc 2025;14:e76009) the authors noted one error.

In Textbox 1, under both forced-choice questions, the first option:

≤1 to 3

Has been revised to:

≤1

And the following option has been added underneath the first option:

1.1 to 3

So that Textbox 1 now reads as follows:

**Forced-choice questions in the survey questionnaire.**

**I anticipate ≥1 physician (possibly, including myself) at my hospital will become ready to use AI to prescribe a drug within _____ years.**

- ≤1
- 1.1 to 3
- 3.1 to 5
- 5.1 to 10
- >10
- Never

**I anticipate I myself will become ready to use AI to prescribe a drug within _____ years.**

- ≤1
- 1.1 to 3
- 3.1 to 5
- 5.1 to 10
- >10
- Never

The correction will appear in the online version of the paper on the JMIR Publications website together with the publication of this correction notice. Because this was made after submission to PubMed, PubMed Central, and other full-text repositories, the corrected article has also been resubmitted to those repositories.

