# Supplementary material for: Factors Affecting the Receptiveness of Chinese Internists and Surgeons Toward Artificial Intelligence–Driven Drug Prescription: Protocol for a Systematic Survey Study
Source: JMIR Res Protoc. 2025 Aug 14;14:e76009. doi: 10.2196/76009 (PMC12395097; doi:10.2196/76009)
Supplement: Multimedia Appendix 1 [file resprot_v14i1e76009_app1.docx]

**Multimedia Appendix 1. Questionnaire (English translation).** ***v.2025-04.en***

| **Category** | **Sub-category** | **Question** |
| --- | --- | --- |
| Personal attributes | Demographic information | Q1. Birth year: ____ |
|  |  | Q2. Sex:  ⬜ Female  ⬜ Male |
|  |  | Q3. Highest educational degree:  ⬜ Associate  ⬜ Bachelor (including bachelor of medicine)  ⬜ Master  ⬜ Ph.D. |
|  |  | Q4. I started practicing clinical medicine in year _____.  On average I see ≈____ out-patients in a week.  On average I am involved in management of ≈____ in-patients in a week. |
|  |  | Q5. Current physician rank:  ⬜ Chief physician (*zhuren yishi*)  ⬜ Associate chief physician (*fuzhuren yishi*)  ⬜ Junior attending physician (*zhuzhi yishi*) |
|  |  | Q6. Clinical specialty:  Internal Medicine  ⬜ General Internal Medicine  ⬜ Cardiology  ⬜ Endocrinology  ⬜ Gastroenterology  ⬜ Hematology & Oncology  ⬜ Infectious Diseases  ⬜ Nephrology  ⬜ Neurology  ⬜ Respiratory  ⬜ Rheumatology  ⬜ Other specialty  Surgery  ⬜ General Surgery  ⬜ Cardiothoracic Surgery  ⬜ Neurosurgery  ⬜ Orthopedics  ⬜ Thoracic Surgery  ⬜ Other specialty |
|  | Knowledge of AI | Q7. I am knowledgeable in AI technology in general (that is, not limited to application of AI in medicine).  ⬜ Strongly agree *I am confident to make decisions regarding AI technology based on my own judgment.*  ⬜ Agree *I can make my own decisions regarding AI technology after consulting with experts and/or perusing literature.*  ⬜ Neutral *If there are experts showing me options for AI technology and explaining to me their pros and cons, I am confident I will make the best technological decision based on the presented options.*  ⬜ Disagree *I am aware of technological trends of AI but I would rather someone else make decisions for me regarding AI technology.*  ⬜ Strongly disagree *I am unfamiliar with technological trends of AI and would rather someone else make decisions for me regarding AI technology.* |
|  |  | Q8. I am knowledgeable in medical AI technology.  ⬜ Strongly agree *I am confident to make decisions regarding the use of medical AI technology based on my own judgment.*  ⬜ Agree *I can make my own decisions regarding the use of medical AI technology after consulting with experts and/or perusing literature.*  ⬜ Neutral *If there are experts showing me options for medical AI technology and explaining to me their pros and cons, I am confident I will make the best technological decision for medical AI based on the presented options.*  ⬜ Disagree *I am aware of technological trends of medical AI but I would rather someone else make decisions for me regarding medical AI technology.*  ⬜ Strongly disagree *I am unfamiliar with technological trends of medical AI and would rather someone else make decisions for me regarding medical AI technology.* |
|  |  | Q9. I am experienced with using AI in work and/or daily life (that is, not limited to application of AI in medicine).  ⬜ Strongly agree *NOT ONLY am I good at using AI in my work and/or daily life BUT ALSO I constantly and actively learn new skills to expand my AI toolbox.*  ⬜ Agree *I use AI in my work and/or daily life AND I am good at using AI tool(s).*  ⬜ Neutral *I have some experience with using AI in my work and/or daily life.*  ⬜ Disagree *I rarely use AI in my work and/or daily life.*  ⬜ Strongly disagree *I rarely use AI in my work and/or daily life AND I would rather I have nothing to do with AI.* |
|  |  | Q10. I am experienced with using medical AI in my clinical practice.  ⬜ Strongly agree *NOT ONLY am I good at using medical AI BUT ALSO I constantly and actively learn new skills to expand my toolbox for medical AI.*  ⬜ Agree *I use medical AI AND I am good at using medical AI tool(s).*  ⬜ Neutral *I have some experience with using medical AI.*  ⬜ Disagree *I rarely use medical AI.*  ⬜ Strongly disagree *I rarely use medical AI AND I would rather I have nothing to do with medical AI.* |
|  | Perception of AI | Q11. I believe AI eventually will transform the healthcare industry.  ⬜ Strongly agree *I believe AI eventually will dramatically transform healthcare AND moreover any physician who refuses to ‘ride the wave’ would become outdated.*  ⬜ Agree *I believe AI eventually will dramatically transform healthcare, but some ‘conventional’ physicians who do not use AI can still find their niche.*  ⬜ Neutral *I believe AI eventually will transform healthcare to some extent, and not all physicians need to learn to use medical AI.*  ⬜ Disagree *I believe there will be limited niche applications of AI in medicine, AND healthcare will continue to rely on ‘conventional’ physicians who do not utilize or depend on medical AI.*  ⬜ Strongly disagree *I believe healthcare will continue to rely on ‘conventional’ physicians who do not utilize or depend on medical AI. I believe medical AI is hyped and, like other hypes before it, it will fail to deliver its promise.* |
|  |  | Q12. I believe AI eventually will augment physicians’ capabilities.  ⬜ Strongly agree *I believe judicious use of medical AI will make most physicians better physicians and better able to care for patients, AND moreover most physicians will better love their jobs because of medical AI.*  ⬜ Agree *I believe judicious use of medical AI will make most physicians better physicians and better able to care for patients.*  ⬜ Neutral *I believe medical AI will augment capabilities of some but not all physicians.*  ⬜ Disagree *For most physicians I believe medical AI will not augment their capability to care for patients.*  ⬜ Strongly disagree *For most physicians I believe medical AI will not augment their capability to care for patients AND moreover, if medical AI becomes common in clinical practice, I believe* *many physicians will not enjoy their jobs as much as they do today.* |
|  |  | Q13. I believe AI eventually will improve healthcare quality.  ⬜ Strongly agree *With judicious use of medical AI, I believe most patients will be better cared for and attain better health AND moreover most patients will be more satisfied with the healthcare they receive.*  ⬜ Agree *With judicious use of medical AI, I believe most patients will be better cared for and attain better health.*  ⬜ Neutral *I believe medical AI will improve the quality or expediency of healthcare for some patients but lower the quality or expediency for others.*  ⬜ Disagree *For most patients, I believe medical AI would not make meaningful and positive impact on their healthcare.*  ⬜ Strongly disagree *For most patients, I believe medical AI would not make meaningful and positive impact on their healthcare AND moreover, if medical AI becomes common in clinical practice, I believe many patients will become less satisfied with the healthcare they receive.* |
|  |  | Q14. I believe AI eventually will improve equity in access to healthcare.  ⬜ Strongly agree *NOT ONLY I believe medical AI will make healthcare more equitable and promote population health, BUT ALSO I believe medical AI is one of the most important tools for achieving common prosperity.*  ⬜ Agree *I believe medical AI will make healthcare more equitable and promote population health.*  ⬜ Neutral *I believe medical AI is only one of the many tools we could try to test whether they could promote population health.*  ⬜ Disagree *I believe medical AI will not make healthcare more equitable AND it will not promote population health.*  ⬜ Strongly disagree *NOT ONLY I believe medical AI will not make healthcare more equitable, BUT ALSO I believe medical AI initiatives will divert crucial resources from other more impactful tools for promoting population health.* |
|  |  | Q15. I believe medical AI eventually will facilitate physicians’ training.  ⬜ Strongly agree *NOT ONLY I believe medical AI eventually will expedite the training of new physicians, BUT ALSO I believe, because of medical AI, new waves of physicians will be better trained compared to trainees in conventional training programs.*  ⬜ Agree *I believe medical AI will expedite the training of new physicians.*  ⬜ Neutral *I believe medical AI will facilitate some but not all aspects of physician training.*  ⬜ Disagree *I believe reliance on medical AI will interfere with junior physicians’ learning journey and slow down their learning.*  ⬜ Strongly disagree *I believe reliance on medical AI will interfere with junior physicians’ learning journey, slow down their learning, AND lead to new waves of under-qualified physicians.* |
|  |  | Q16. When considering medical AI, which of the following is more important to me? Choose one.  ⬜ A medical AI system is efficacious in delivering what it promises.  ⬜ A medical AI system does not affect my autonomy as a physician. |
|  | Perception of AI prescription | Q17. I think AI prescription might be useful in the following situations: (Choose ≤3 options that best describe what I think.)  ⬜ When clinical guidelines clearly specify standardized treatment plans.  ⬜ When refilling drug prescription that is already known to be safe in a given patient.  ⬜ When there are too many clinical factors to consider before making a decision on drug prescription.  ⬜ When it is an unusual occasion wherein total reliance on humans may cause delay in starting therapy or lapse in decision-making.  ⬜ When there is shortage of qualified staffs.  ⬜ When physicians need to focus on more important clinical decisions.  ⬜ When the AI system explains the rationale underlying the prescription it recommends.  ⬜ None of the above. |
| Perceived importance of technological attributes | Vetted efficacy | Q18. Which of the following aspects of efficacy-vetting are the most important for accepting the use of an AI prescription model? (Choose ≤2 options that best describe what I think.)  ⬜ The AI model was developed using data from cohorts that are similar to my patients.  ⬜ The model has been validated in ≥1 independent cohort.  ⬜ The model has been validated in ≥1 domestic cohort.  ⬜ The model’s test result has been published in a highly-regarded peer review journal.  ⬜ The model is endorsed by ≥1 reputable international medical professional society.  ⬜ The model is endorsed by ≥1 reputable domestic medical professional society. |
|  | Expediency | Q19. How would I define ‘expediency’ of an AI prescription model? (Choose ≤3 options that best describe what I think.)  ⬜ The model can be embedded in the workflow of my clinical practice.  ⬜ The model would not increase my work load.  ⬜ The model would enable me to grow my clinical practice while maintaining quality of care.  ⬜ The model would enable me to shorten my work hours while maintaining quality of care.  ⬜ The model would allow me to focus on other activities that matter.  ⬜ The model could help me mitigate the risk of making errors.  ⬜ The model could augment my capability for decision-making.  ⬜ The model would improve patients’ trust in me.  ⬜ The model would make it easier for me to hire and train junior physicians. |
|  | Transparency | Q20. Before I decide to use an AI prescription model in my clinical practice, technical details regarding how the model was built and validated (even if not everyone can understand them) need to be transparent.  ⬜ Strongly agree *Before I can decide whether to use an AI prescription model in my clinical practice, I personally need to know all the technical details related to model construction including training data, model-fitting, performance metrics, and validation data.*  ⬜ Agree *I only need to know a portion of technical details related to model construction before I decide whether to use the model in my clinical practice.*  ⬜ Neutral *Whether the technical details of AI model construction are accessible to me is irrelevant to my decision on whether to adopt an AI prescription model in my clinical practice.*  ⬜ Disagree *Whether the technical details of AI model construction are accessible to me is irrelevant to my decision on whether to adopt an AI prescription model in my clinical practice. All I need to know is that the AI prescription model has been vetted by experts, and there is NO need for the model’s technical details to be made fully transparent after review by experts.*  ⬜ Strongly disagree *Whether the technical details of AI model construction are accessible to me is irrelevant to my decision on whether to adopt an AI prescription model in my clinical practice. All I need to know is that the AI prescription model has been vetted by experts, and there is NO need for the model’s technical details to be made fully transparent after review by experts. MOREOVER, I believe mandatory full disclosure of proprietary information might discourage innovation and harm societal progress.* |
|  | Explainability | Q21. Which of the following aspects of an AI prescription model’s ‘explainability’ are the most important to me? (Choose ≤2 options that best describe what I think.)  ⬜ I know and agree with the model’s input variables.  ⬜ I understand how the AI model converts input variables to prescriptions.  ⬜ I am able to explain to my colleagues how the model works.  ⬜ I am able to explain to my patients how the model works.  ⬜ When the AI model’s recommendation is discordant with my opinion, the model can explain to me its reasoning. |
|  | Governance/  stewardship | Q22. Which of the following aspects of governance/stewardship of AI prescription are the most important to me? (Choose ≤2 options that best describe what I think.)  ⬜ There is a trustworthy mechanism for the model’s maintenance and/or upgrades.  ⬜ I know whom to contact when I encounter problem(s) and/or need help with the AI prescription model.  ⬜ There is a trustworthy mechanism for monitoring quality, bias, and safety of AI prescriptions.  ⬜ There is an auditing mechanism for upholding the AI model’s adherence to data privacy and security. |
|  | Trade-off | Q23. Which of the following technological attributes of an AI prescription model are the most important to me? (Rank the following items from the most important [rank = 1] to the least important [rank = 5].)  ⬜ Vetted efficacy.  ⬜ Expediency.  ⬜ Transparency.  ⬜ Explainability.  ⬜ Governance/stewardship. |
| Perceived importance of institutional attributes | Culture | Q24. In my opinion, which of the following aspects of institutional culture would be the most important for successful adoption of AI prescription at my institution? (Choose ≤4 options that best describe what I think.)  ⬜ My hospital values innovation.  ⬜ My hospital embraces new technologies.  ⬜ My hospital is willing to invest resources in AI initiatives.  ⬜ My hospital values employee satisfaction and interpersonal harmony.  ⬜ My hospital is team-oriented and values collaboration.  ⬜ My hospital upholds transparent communications to employees.  ⬜ My hospital promotes learning and talent development.  ⬜ My hospital focuses on achieving measurable goals and celebrates success.  ⬜ My hospital values stability and continuity.  ⬜ My hospital is receptive to changes of workflows. |
|  | Change management | Q25. In my opinion, adoption of AI prescription at my institution would be more likely to be successful if _____ (Choose ≤3 options that best describe what I think.)  ⬜ The leadership team at my hospital supports the adoption of AI in prescription.  ⬜ My hospital provides guidance on the use of AI in prescription.  ⬜ There would be a dedicated team of local champions to ensure successful implementation of AI prescription models.  ⬜ Planning of the AI prescription initiative is transparent.  ⬜ Planning of the AI prescription initiative involves a multi-disciplinary team.  ⬜ Planning of the AI prescription initiative consults or involves me.  ⬜ There is a plan for educating and training the staffs on the AI prescription model’s use. |
|  | IT and data science | Q26. In my opinion, which of the following aspects of information technology (IT) and data science resource availability and/or access are the most important for successful adoption of AI prescription at my institution? (Rank the following items from the most important [rank = 1] to the least important [rank = 3].)  ⬜ A reliable and capable in-house IT/data science specialist team.  ⬜ A reliable and capable 3^rd^-party partner(s) or vendor(s).  ⬜ Employees from disciplines other than IT/data science are also held responsible for successful introduction of the new technology. |
|  | Differentiation | Q27. In my opinion, after successful adoption of AI prescription, my institution will need to improve _____ to maintain or further its edge compared to other medical institutions. (Choose ≤3 options that best describe what I think.)  ⬜ Throughput.  ⬜ Cost-effectiveness.  ⬜ Empathy and caregiving.  ⬜ Ability of specialists from multiple disciplines to work collaboratively to deliver patient care.  ⬜ Capability to care for patients who have special needs.  ⬜ Expertise to manage diseases that are difficult to treat.  ⬜ Integration of clinical practice with research to accelerate the advent of next-generation treatments. |
| Perceived importance of governmental attributes | Nation-wide commitment | Q28. In my opinion, which of the following is the most important for successful adoption of AI prescription in my clinical practice? (Rank the following items from the most important [rank = 1] to the least important [rank = 3].)  ⬜ There is a nation-wide initiative to promote the use of AI in healthcare.  ⬜ There is a nation-wide initiative to improve technological infrastructure for medical AI.  ⬜ There is a nation-wide initiative to educate/train talents for medical AI. |
|  | AI standards | Q29. In my opinion, who should set the standards for AI prescription systems? Choose one that best applies.  ⬜ Regulatory authorities.  ⬜ Professional medical societies.  ⬜ Each healthcare organization should set its own standard.  ⬜ Regulatory authorities + Professional medical societies  ⬜ Regulatory authorities + Each healthcare organization  ⬜ Professional medical societies + Each healthcare organization  ⬜ Regulatory authorities + Professional medical societies +  Each healthcare organization |
|  | Remuneration policy | Q30. In my opinion, who should be remunerated when an AI model is utilized for prescribing a drug (that is, additional charge beyond drug price)? Choose one that best applies.  ⬜ No one.  ⬜ The hospital who has invested in the new AI technology.  ⬜ The physician who uses the model and reviews its output.  ⬜ The developer who built and maintains/updates the model.  ⬜ Hospital + Physician  ⬜ Hospital + Developer  ⬜ Physician + Developer  ⬜ Hospital + Physician + Developer |
| To sum up |  | Q31. I anticipate ≥1 physician (possibly including myself) at my hospital will become ready to utilize AI to prescribe a drug within _____ years.  ⬜ ≤1  ⬜ 1.1 – 3  ⬜ 3.1 – 5  ⬜ 5.1 – 10  ⬜ >10  ⬜ Never |
|  |  | Q32. I anticipate I myself will become ready to utilize AI to prescribe a drug within _____ years.  ⬜ ≤1  ⬜ 1.1 – 3  ⬜ 3.1 – 5  ⬜ 5.1 – 10  ⬜ >10  ⬜ Never |
