# Supplementary material for: Factors Affecting the Receptiveness of Chinese Internists and Surgeons Toward Artificial Intelligence–Driven Drug Prescription: Protocol for a Systematic Survey Study
Source: JMIR Res Protoc. 2025 Aug 14;14:e76009. doi: 10.2196/76009 (PMC12395097; doi:10.2196/76009)
Supplement: Multimedia Appendix 2 [file resprot_v14i1e76009_app2.docx]

**Multimedia Appendix 2. Questionnaire (original Chinese version). *v.2025-04.zh***

| **第一级类别** | **第二级类别** | **问题** |
| --- | --- | --- |
| 个人基本情况 | 人口学信息 | Q1. 我出生于公元 ____ 年。 |
|  |  | Q2. 我的性别：  ⬜ 男  ⬜ 女 |
|  |  | Q3. 我的最高学历：  ⬜ 专科  ⬜ 本科（包括医学学士）  ⬜ 硕士  ⬜ 博士 |
|  |  | Q4. 我于公元 _____ 年开始从事临床实践工作。  我每周在门诊（包含不是我本人的门诊）看的患者平均人数：约 ____ 个人。  我每周负责或参与治疗的住院患者平均人数：约 ____ 个人。 |
|  |  | Q5. 我的专业职称：  ⬜ 主任医师  ⬜ 副主任医师  ⬜ 主治医师 |
|  |  | Q6. 我的临床专业领域：  内科  ⬜ 内科全科  ⬜ 心脏内科  ⬜ 内分泌科  ⬜ 消化内科  ⬜ 肿瘤内科或血液科  ⬜ 感染性疾病科  ⬜ 肾脏内科  ⬜ 神经内科  ⬜ 呼吸内科  ⬜ 风湿与免疫科  ⬜ 其它  外科  ⬜ 普通外科  ⬜ 心脏外科  ⬜ 胸腔外科  ⬜ 神经外科  ⬜ 骨外科  ⬜ 其它 |
|  | 自评个人的AI知识水平 | Q7. 我对AI技术整体上较为了解（不限于医学领域中的AI应用）  ⬜ 非常同意*（我有信心根据自己的判断，对AI技术的使用做出决策。）*  ⬜ 同意*（在咨询专家或查询文献后，我能够自己对AI技术的使用做出决策。）*  ⬜ 中立*（如果有专家向我展示不同的AI技术选项，并解释它们各自的利弊，我相信我能从专家提供给我的多个AI技术选项中挑选出最佳的技术方案。）*  ⬜ 不同意*（我知道AI的技术趋势，但我宁愿他人帮我做AI技术相关的决策。）*  ⬜ 非常不同意*（我不熟悉AI的技术趋势，我宁愿他人帮我做AI技术相关的决策。）* |
|  |  | Q8. 我对医疗AI的技术较为了解  ⬜ 非常同意*（我有信心根据自己的判断，对医疗AI技术的使用做出决策。）*  ⬜ 同意*（在咨询专家或查询文献后，我能够自己对医疗AI技术的使用做出决策。）*  ⬜ 中立*（如果有专家向我展示不同的医疗AI技术选项，并解释它们各自的利弊，我相信我能从专家提供给我的多个医疗AI技术选项中挑选出最佳的技术方案。）*  ⬜ 不同意*（我知道医疗AI技术的发展趋势，但我宁愿他人帮我做医疗AI技术相关的决策。）*  ⬜ 非常不同意*（我不熟悉医疗AI的技术趋势，我宁愿他人帮我做医疗AI技术相关的决策。）* |
|  |  | Q9. 我在工作和/或日常生活中有使用AI的经验（不限于医学应用）  ⬜ 非常同意*（在日常工作和/或生活中，我不仅擅长使用AI工具，且我持续主动学习新技术来充实我的AI工具包。）*  ⬜ 同意*（我在日常工作和/或生活中使用AI工具且我擅长使用AI工具。）*  ⬜ 中立*（我在日常工作和/或生活中有一些使用AI工具的经验。）*  ⬜ 不同意*（我在日常工作和/或生活中很少使用AI工具。）*  ⬜ 非常不同意*（我在日常工作和/或生活中很少使用AI工具，且我宁愿自己不需使用AI。）* |
|  |  | Q10. 我在自己的临床实践中有使用医疗AI的经验  ⬜ 非常同意*（我在自己的临床实践中不仅熟练使用医疗AI工具，且我持续主动学习新技术来充实我的医疗AI工具包）*  ⬜ 同意*（我在自己的临床实践中使用医疗AI工具且我擅长使用医疗AI工具。）*  ⬜ 中立*（我在自己的临床实践中有一些使用医疗AI工具的经验。）*  ⬜ 不同意*（我在自己的临床实践中很少使用医疗AI工具。）*  ⬜ 非常不同意*（我在自己的临床实践中很少使用医疗AI工具，且我宁愿自己不需使用医疗AI。）* |
|  | 自评个人对AI的看法 | Q11. 我相信AI最终会给医疗行业带来变革。  ⬜ 非常同意*（我相信AI最终会给医疗行业带来巨大的变革，且我认为拒绝抓住AI机遇的医生将被时代淘汰。）*  ⬜ 同意*（我相信AI最终会给医疗行业带来巨大的变革；但我也认为，在一些场合不使用AI不会影响医生胜任工作。）*  ⬜ 中立*（我相信AI最终会给医疗行业带来某种程度的变革，而不是所有的医生都需要学习使用医疗AI。）*  ⬜ 不同意*（我相信，最终医疗AI的可应用领域有限，且医疗行业仍将持续依赖于不使用AI的医生。）*  ⬜ 非常不同意*（我相信医疗行业仍将持续依赖于不使用AI的医生，且我相信“医疗AI”仅是一种炒作，最终不能达到它所宣称预期目标。）* |
|  |  | Q12. 我相信AI最终会提升医生医疗能力。  ⬜ 非常同意*（我相信合理使用医疗AI终将使大部分医生更好地照护患者，且医疗AI将使大部分医生更热爱自己的工作。）*  ⬜ 同意*（我相信合理使用医疗AI终将使大部分医生更好地照护患者。）*  ⬜ 中立*（我相信医疗AI能提高部分医生照护患者的能力。）*  ⬜ 不同意*（我相信，对于大部分的医生，照护患者的能力不会因为医疗AI而提升。）*  ⬜ 非常不同意*（我相信，对于大部分的医生，照护患者的能力不会因为医疗AI而提升。此外，如果医疗AI的临床应用越来越普遍，我认为许多医生会因此降低自己对工作的热情。）* |
|  |  | Q13. 我相信AI最终能提升健康照护质量。  ⬜ 非常同意*（我相信合理使用医疗AI能使大部分患者得到更好的照护，提升他们的健康，且我相信大多数患者将对他们获得的医疗照护更满意。）*  ⬜ 同意*（我相信合理使用医疗AI能使大部分患者得到更好的照护，提升他们的健康。）*  ⬜ 中立*（我相信医疗AI能提升一部分患者所获得的医疗照护，但会降低另一部分患者所获得的医疗照护。）*  ⬜ 不同意*（我相信，对于大部分患者的医疗照护，医疗AI的正面影响有限。）*  ⬜ 非常不同意*（我相信，对于大部分患者的医疗照护，医疗AI的正面影响有限。此外，如果医疗AI的临床应用越来越普遍，我认为许多患者将不满于他们获得的医疗照护。）* |
|  |  | Q14. 我相信AI最终能提升健康照护的公平性。  ⬜ 非常同意*（我相信医疗AI终将使医疗照护更加公平并提升公共健康水平，且我相信医疗AI是实现社会共同富裕的重要手段之一。）*  ⬜ 同意*（我相信医疗AI能使医疗照护更加公平并提升公共健康水平。）*  ⬜ 中立*（我相信医疗AI只是可能促进公共健康水平的众多手段之一而已，它仍待验证。）*  ⬜ 不同意*（我认为我们终将证实医疗AI不能使医疗照护更加公平或提升公共健康水平。）*  ⬜ 非常不同意*（我认为我们终将证实医疗AI不能使医疗照护更加公平或提升公共健康水平，且我认为投入医疗AI是浪费关键资源，剥夺了其它更可能促进公共健康的措施的成功机会。）* |
|  |  | Q15. 我相信医疗AI最终能使医生培养更加便捷。  ⬜ 非常同意*（我相信在未来，医疗AI能加速新医生的培养，且我相信，如果训练体系涵括医疗AI，医生将能被训练得更好。）*  ⬜ 同意*（我相信在未来，医疗AI能加速新医生的培养。）*  ⬜ 中立*（我相信在未来，医疗AI能加速新医生在某些方面的培养。）*  ⬜ 不同意*（我相信依赖医疗AI将阻碍初级医生的成长历练，甚至减缓他们的学习速度。）*  ⬜ 非常不同意*（我相信依赖医疗AI将阻碍初级医生的成长历练，减缓他们的学习速度，且产生一批不合格的新医生。）* |
|  |  | Q16.下面哪个您认为更重要？（单选）  ⬜ 医疗AI实现其预期诊疗效益。  ⬜ 医疗AI不影响我本人作为医生的自主性。 |
|  | 个人对AI处方的看法 | Q17. 我认为AI处方在以下哪些情境中可能是有用途的：（请选择最符合我观点的选项，最多3项）  ⬜ 当临床指南明确规范了标准化的治疗方案时。  ⬜ 当为某位患者延续开具相同处方时（已知该处方对于该患者是安全的）。  ⬜ 当在决定药物处方前需要考虑太多临床因素时。  ⬜ 当在某些特殊状况，完全依赖于人的决策可能会造成治疗延迟或决策失效时。  ⬜ 当合格员工短缺即人手不足时。  ⬜ 当医生需将精力聚焦于更重要的其他临床决策时。  ⬜ 当AI模型能说明为何它推荐某个处方（即解释该推荐处方的背后原理）时。  ⬜ 以上皆非。 |
| AI模型的 技术层面 | 模型有效性的认证 | Q18. 下面关于AI处方有效性认证的描述，哪些对我接受使用AI处方模型最重要？  （选择最能代表我想法的选项，最多2项）  ⬜ 研究者开发模型时所运用的患者队列类似于我的患者。  ⬜ 模型已经在≥1个独立队列验证。  ⬜ 模型已经在中国患者验证。  ⬜ 模型测试的结果已经刊在高水平的学术期刊。  ⬜ 有声望的国际医学专家协会认可该模型。  ⬜ 有声望的中国医学专家协会认可该模型。 |
|  | 模型实用性 | Q19. 我如何定义AI处方模型的“实用性”？（选择最符合我想法的选项，最多3个）  ⬜ 模型能被整合到我的临床工作流程。  ⬜ 模型不会增加我的工作负担。  ⬜ 在保证患者照护质量的前提下，模型能扩大我的诊疗量。  ⬜ 在保证患者照护质量的前提下，模型能缩短我的工作时间或使得我的工作更轻松。  ⬜ 模型让我能将精力聚焦于其它更重要的事情上。  ⬜ 模型能减少我的犯错风险。  ⬜ 模型能增强我的临床决策能力。  ⬜ 模型能提升患者对我的信任。  ⬜ 模型能帮助培训资浅医生，使我更容易扩充团队。 |
|  | 模型透明性 | Q20. 在我决定使用AI处方模型之前，模型建立和模型验证的技术细节应是透明的（即使不是每个人都能理解这些技术细节）。  ⬜ 非常同意*（在我考虑我自己是否将在临床实践中使用AI处方模型时，我本人需要知道关于模型构建的所有技术细节，包括训练数据、模型拟合演算法、模型评价方法和验证数据。）*  ⬜ 同意*（在我考虑我自己是否将在临床实践中使用AI处方模型时，我本人仅需要知道关于模型构建的部分技术细节。）*  ⬜ 中立*（在我考虑我自己是否将在临床实践中使用AI处方模型时，我本人不需要知道模型构建的技术细节。）*  ⬜ 不同意*（在我考虑我自己是否将在临床实践中使用AI处方模型时，我本人不需要知道模型构建的技术细节。既然AI处方模型已被专家审查过，我认为没有必要在通过审查之后强制要求将模型的技术细节完全透明化。）*  ⬜ 非常不同意*（在我考虑我自己是否将在临床实践中使用AI处方模型时，我本人不需要知道模型构建的技术细节。既然AI处方模型已被专家审查过，我认为没有必要在通过审查之后强制要求将模型的技术细节完全透明化。事实上，我认为强制要求将技术秘密完全公开可能会阻碍科技创新，并损害社会进步。）* |
|  | 模型可解释性 | Q21. 下面哪些关于AI处方模型‘可解释性’的描述对我最重要？（选择最符合我想法的选项，最多2个）  ⬜ 我了解并认可模型所使用的输入变量。  ⬜ 我理解AI模型如何将输入变量转换成模型推荐的药物处方。  ⬜ 我能够向我的同事解释模型是如何运作的。  ⬜ 我能够向我的病人解释模型是如何运作的。  ⬜ 当AI模型推荐的药物处方与我的观点不一致时，模型能够向我解释它为什么给出那样的建议。 |
|  | 监督/管理 | Q22. 下面哪些关于AI处方模型监督/管理的描述对我最重要？（选择最符合我想法的选项，最多2个）  ⬜ 有可信赖的机制来维护AI处方模型和/或升级AI处方模型。  ⬜ 当使用AI处方模型遇到问题或需要帮助时，我知道联系谁。  ⬜ 有可信赖的机制来监督AI处方的质量、偏倚和安全性。  ⬜ 有审查机制来监督AI模型遵守数据隐私和数据安全的相关规定。 |
|  | 多个维度之间的重要性权衡 | Q23. 下面关于AI处方模型的属性对我的重要性排序？（将下列选项按重要性由高［标“①”］到低［标“⑤”］排序。）  ⬜ 有效性。  ⬜ 实用性。  ⬜ 透明性。  ⬜ 可解释性。  ⬜ 监督/管理模式。 |
| 医院属性 | 医院内部文化 | Q24. 我认为，以下哪些医院内部文化因素对于能在医院里成功应用AI处方模型至关重要？ （选择最符合我想法的选项，最多4个）  ⬜ 我的医院重视创新。  ⬜ 我的医院拥抱新技术。  ⬜ 我的医院愿意为AI投注资源。  ⬜ 我的医院重视员工满意度和人际和谐。  ⬜ 我的医院以团队为导向且重视合作。  ⬜ 我的医院注重透明沟通。  ⬜ 我的医院关注员工的学习和技能成长。  ⬜ 我的医院专注于达成具体目标。  ⬜ 我的医院重视稳定和延续性。  ⬜ 我的医院有弹性，愿意变更工作流程。 |
|  | 医院推动变革的机制 | Q25. 我认为在以下哪种情境，医院更能成功应用AI处方模型？（选择最符合我想法的选项，最多3个）  ⬜ 我的医院领导团队支持运用AI处方模型。  ⬜ 我的医院有关于AI处方模型的发展战略。  ⬜ 我的医院内部有项目团队确保AI处方模型成功落地。  ⬜ 我的医院内部采用AI处方模型的规划过程是透明的。  ⬜ 我的医院内部采用AI处方模型的规划是由多学科团队群策群力。  ⬜ 我的医院内部采用AI处方模型的规划有我个人参与。  ⬜ 我的医院对员工使用AI处方模型有教育和培训计划。 |
|  | 信息技术和数据科学 | Q26. 我认为以下信息技术和数据科学资源的相关因素对在医院内成功应用AI处方模型最重要？（对下列选项按重要性从高［标“①”］到低［标“③”］进行排序）  ⬜ 我的医院内部有专属的信息与数据科学员工团队。  ⬜ 关于信息与数据科学，我的医院选择可靠的第三方合作伙伴或供应商。  ⬜ 在我的医院引进AI处方模型时，不全是信息与数据科学专家承担成败责任，其他学科领域的员工也需承担任务和承担等同的成败责任。 |
|  | 差异化发展 | Q27. 我认为在成功应用AI处方模型后，医院需要在哪些方面继续强化以保持或进一步提升优势？（选择最符合我想法的选项，最多3个）  ⬜ 提升就诊量。  ⬜ 提升医院的成本效益。  ⬜ 提升基于同理心的关怀供给。  ⬜ 强化多学科专家的协作能力以提升患者照护水平。  ⬜ 提升对特殊情况患者的照护能力。  ⬜ 提升治疗难治疾病的能力。  ⬜ 提升将临床实践与研究相结合的能力，加速开发新一代治疗方案。 |
| 大环境 | 政府引导 | Q28. 我认为下面哪些描述对我本人在临床实践中成功应用AI最重要？（对下列选项按重要性从高[标“①”]到低[标“③”]进行排序）  ⬜ 政府有政策导向推动在医疗照护中使用AI。  ⬜ 政府大力改进医疗AI技术所需的基础设施。  ⬜ 政府大力开展医疗AI所需专业技术人才的教育与培养。 |
|  | AI标准 | Q29. 我认为AI处方模型的应用标准应该由谁来制定？（选择一个最符合我想法的选项）  ⬜ 政府管理机构  ⬜ 医学专家协会  ⬜ 每个医疗机构设定自己的标准  ⬜ 政府管理机构 + 医学专家协会  ⬜ 政府管理机构 + 每个医疗机构设定自己的标准  ⬜ 医学专家协会 + 每个医疗机构设定自己的标准  ⬜ 政府管理机构 + 医学专家协会 + 每个医疗机构设定自己的标准 |
|  | 给付政策 | Q30. 每当AI模型开具一个药物处方时，我认为谁应被支付技术手续费（即药物费用之外的额外费用）？（选择一个最符合我想法的选项）  ⬜ 没有人（不应存在技术手续费）  ⬜ 投资应用AI处方技术的医院  ⬜ 运用AI处方模型并核实模型输出结果的医生  ⬜ 开发和维护模型的专业模型团队  ⬜ 医院 + 医生  ⬜ 医院 + 模型团队  ⬜ 医生 + 模型团队  ⬜ 医院 + 医生 + 模型团队 |
| 最终 灵魂拷问 |  | Q31. 我预期，在我的医院，有≥1个医生（可能包含我自己）将在_____年内会愿意开始运用AI开处方。(选择一个最符合我想法的选项)  ⬜ ≤1 年  ⬜ 1.1 – 3 年  ⬜ 3.1 – 5 年  ⬜ 5.1 – 10 年  ⬜ >10 年  ⬜ 永远不会 |
|  |  | Q32. 我预期，我本人在_____年内会愿意开始运用AI开处方。(选择一个最符合我想法的选项)  ⬜ ≤1 年  ⬜ 1.1 – 3 年  ⬜ 3.1 – 5 年  ⬜ 5.1 – 10 年  ⬜ >10 年  ⬜ 永远不会 |
